# High Copy Number Variations Correlate with a Pro-Tumoral Microenvironment and Worse Prognosis in Acral Lentiginous Melanoma

**DOI:** 10.3390/ijms26094097

**Published:** 2025-04-25

**Authors:** Inés de la Rosa, Pol Sisó, Christopher Ríos, Judith Gracia, Dolors Cuevas, Oscar Maiques, Núria Eritja, Xavier Soria, Joan Angel-Baldó, Sonia Gatius, Lidia Sanchez-Moral, Maria-Rosa Sarrias, Xavier Matias-Guiu, Rosa M. Martí, Anna Macià

**Affiliations:** 1 Oncologic Pathology Group, Institut de Recerca Biomèdica de Lleida (IRBLleida), University of Lleida, 25198 Lleida, Spain; inesrosazurera@gmail.com (I.d.l.R.); polsiso95@gmail.com (P.S.); christopher.rios@udl.cat (C.R.); jgracia@irblleida.cat (J.G.); mcuevas@gss.cat (D.C.); neritja@irblleida.cat (N.E.); sgatius.lleida.ics@gencat.cat (S.G.); fjmatiasguiu.lleida.ics@gencat.cat (X.M.-G.); 2 Department of Pathology and Molecular Genetics, Hospital Universitari Arnau de Vilanova de Lleida, Institut de Recerca Biomèdica de Lleida (IRBLleida), University of Lleida, 25198 Lleida, Spain; 3 Centre of Biomedical Research on Cancer (CIBERONC), Instituto de Salud Carlos III (ISCIII), 28029 Madrid, Spain; 4 Cytoskeleton and Cancer Metastasis Group, The Breast Cancer Now Toby Robins Research Centre, The Institute of Cancer Research, London SM2 5NG, UK; o.m.carlos@qmul.ac.uk; 5 Center for Cancer Biomarkers and Biotherapeutics, Barts Cancer Institute, Queen Mary University of London, John Vane Science Centre, London EC1M 6BQ, UK; 6 Department of Dermatology, Hospital Universitari Arnau de Vilanova de Lleida, Institut de Recerca Biomèdica de Lleida (IRBLleida), University of Lleida, 25198 Lleida, Spain; soriaxavier@gmail.com (X.S.); joan.an.ba@gmail.com (J.A.-B.); 7 Innate Immunity Group, Germans Trias i Pujol Research Institute (IGTP), 08916 Badalona, Spain; lsanchez@igtp.cat (L.S.-M.); mrsarrias@igtp.cat (M.-R.S.); 8 Center for Biomedical Research in Hepatic and Digestive Diseases (CIBERehd), 28029 Madrid, Spain

**Keywords:** acral lentiginous melanoma, copy number variation, tumor microenvironment, secretome, prognosis

## Abstract

Acral lentiginous melanoma (ALM) is a rare melanoma subtype primarily located in acral regions. However, ALMs exhibit a distinctive genetic profile characterized by a high number of copy number variations (CNVs) and limited point mutations. Late diagnosis and restricted therapeutic efficacy contribute to its poor prognosis. The secretome within the tumor microenvironment (TME) influences immune modulation and plays a vital role in melanoma progression. We aim to analyze the role of ALM secretome and CNVs profile with prognosis in primary ALM patients. Here, we demonstrated that high CNV burden (CNVsHigh) was associated with worse clinicopathological characteristics and poor prognosis. Furthermore, our study also revealed that conditioned media (CM) of CNVsHigh genetic profile ALM cell line was associated with pro-tumoral, pro-angiogenic, and immunosuppressive secretome profiles. In addition, CM of CNVsHigh cell lines in vitro promotes macrophage polarization to immunosuppressive phenotype. Moreover, we observed an increased presence of immunosuppressive tumor-associated macrophages (TAMs) at the invasive front (IF) of CNVsHigh ALM biopsies. This research reveals the adverse prognostic impact of CNVsHigh in ALM patients, establishing a novel link with a pro-tumor secretome, offering potential biomarkers for prognosis and personalized treatment to enhanced disease monitoring in ALM patients.

## 1. Introduction

Acral lentiginous melanoma (ALM) is a rare and aggressive melanoma subtype that arises in acral regions, including palms, soles, and nail beds. In contrast to other cutaneous melanomas, which often develop in sun-exposed skin, ALM occurs in areas that are partially or entirely protected from environmental ultraviolet (UV) radiation [1,2]. ALM has a distinctive genetic landscape compared with other cutaneous melanomas, characterized by a high number of copy number variations (CNVs), altering the dosage of oncogenes and tumor suppressor genes, and a low burden of point mutations. BRAF and NRAS are the most commonly mutated oncogenes in acral melanomas, though they occur less frequently than in sun-exposed melanomas, while KIT mutations are more prevalent in ALM. CNV is a well-established feature of ALM, which frequently involves chromosomes 5p, 11q, 12q, and 22q. These genomic regions lead to abnormal regulation of various pathways involved in cell cycle progression, tumor invasion, and gene expression. Key alterations include the amplification of *CDK4*, *CCND1*, *MDM2*, *NOTCH2*, *CRKL*, and *TERT*, among other genes [3,4,5,6,7,8]. Also, this melanoma subtype is associated with poor prognosis, which can be attributed to delayed diagnosis and the limited efficacy of current treatment approaches [9,10,11]. Furthermore, the underlying pathogenesis of this melanoma subtype remains poorly understood [12]. Understanding the genetic landscape of ALM is critical since underlying genetic alterations not only could contribute to tumor initiation but also could influence tumor progression and prognosis and facilitate the selection of target therapies or immunotherapies [7,8,13,14,15].

Tumor microenvironment (TME) has gained significant attention in recent years as a critical determinant of tumor behavior and therapeutic response [16,17]. The intricate interplay between tumor cells, immune cells, and the surrounding stroma in the TME has been shown to influence disease progression and metastases, particularly in tumors with poor prognosis [18]. Although melanoma is a highly immunogenic tumor, during progression, it develops an immunosuppressive mechanism to avoid detection and destruction by innate and adaptive immunity [19,20]. ALM exhibits an especially suppressive tumor immune microenvironment (TIME) with a low proportion of tumor-infiltrating lymphocytes (TILs), in contrast to melanomas originating from non-acral skin [21,22]. Within this complex microenvironment, tumor-associated macrophages (TAMs) have been implicated in promoting tumor growth, invasion, angiogenesis, and immunosuppression [23,24]. TAMs exhibit remarkable plasticity and can display a spectrum of activation states, ranging from the anti-tumor phenotype to immunosuppressive and pro-tumoral phenotype [25,26]. Moreover, the secretome, comprising diverse factors secreted by tumor cells and the surrounding cells, plays a vital role in shaping the TME [27]. The secreted factors contribute to immune modulation, angiogenesis, extracellular matrix remodeling, and communication between different cell types within the TME [27,28]. Elucidating the intricate relationships within the secretome and its impact on ALM progression is crucial for monitoring the disease prognosis, identifying new therapeutic targets and developing personalized treatment strategies [29,30].

Here, we performed a CNV profile characterization in biopsies from patients with primary ALM and assessed the prognostic significance of these genetic alterations. Furthermore, our study aimed to analyze the secretome of ALM cells according to the CNV genetic profile within macrophage polarization in TME. Through integrating secretome analysis with genetic profile, we will be able to identify valuable biomarkers for disease monitoring and the development of novel therapeutic strategies in ALM patients.

## 2. Results

### 2.1. Clinicopathological Characteristics

Our cohort comprised 33 patients with primary ALM diagnosed during the period 2008–2021. 45.5% were male and 54.5% female. Median age was 75 years (Interquartile Range; IQR: 54–83), considering both sexes. Concerning the primary tumor location, 75.8% arise on the foot, and 44% of them are on the soles. Additionally, 75% of hand primary tumors presented a subungual location. The mean Breslow index was 4.2 mm (±3.9 (SD)), and the median was 2.7 mm (IQR: 0.89–7.9). Ulceration was present in 60.6% of biopsies, and the mitotic index was equal to or greater than 1 mitosis per mm^2^ in 63.6% of ALM patients (Table 1).

Concerning the risk associated with AJCC stage, 15.1% of patients presented melanoma in situ (stage 0), 21.2% had low risk (stage IA-IB), 9.1% had intermediate risk (stage IIA), 45.5% had high risk (stage IIB to IIIC), and 9.1% presented distant metastasis at the time of diagnosis (stage IV). At the end of the study, 39.4% of the patients had died due to ALM, 48.4% were alive, and 36.3% had experienced at least one relapse (metastasis) during the study period (Table 1).

### 2.2. Copy-Number Variations Landscape in ALM Patient Biopsies

**Figure 1:**
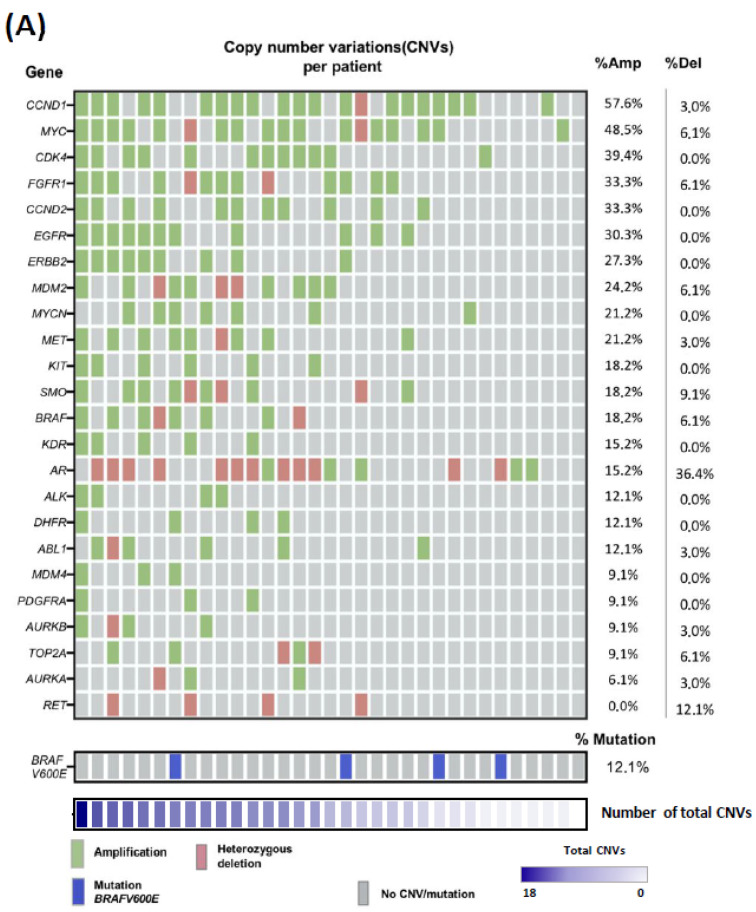

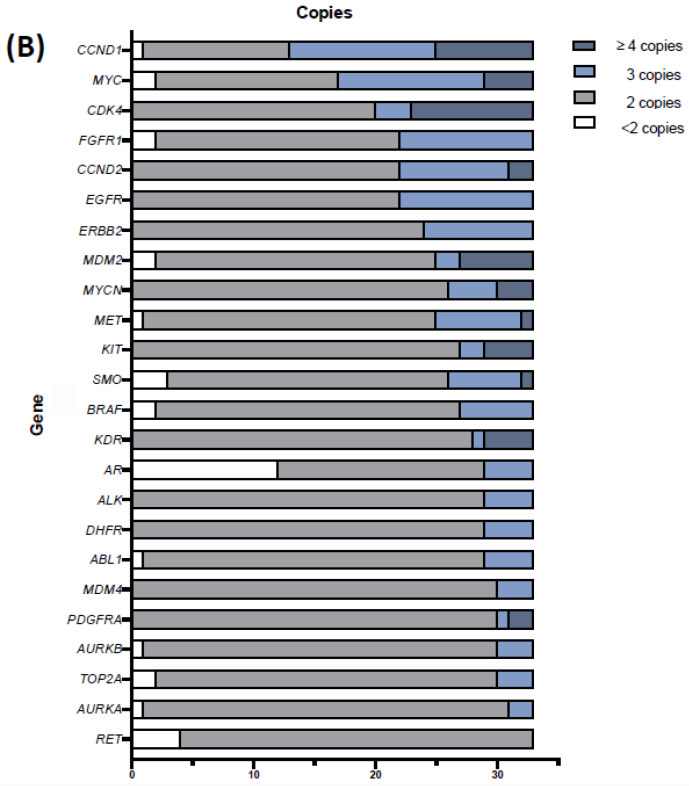
Copy number variations (CNVs) and *BRAFV600E* mutation analysis using Multiplex Ligation-dependent Probe Amplification (MLPA) in biopsies from ALM patients. (**A**) Heatmap of CNVs identified in 24 oncogenes (up) and *BRAFV600E* mutation (bottom), along with the amplification and deletion frequency per gene (right). Genes are arranged in descending order according to the percentage of amplification and number of total CNVs (bottom). (**B**) Copy number quantification of each oncogene analyzed using MLPA. Patients are grouped according to the copy number in the following categories: <2 copies (deletion), 2 copies (diploid), 3 copies (gain of 1 copy), and ≥4 copies. N = 33 patients.

To identify CNVs in biopsies from ALM patients, we used a pre-designed MLPA to detect 24 oncogenes, which are commonly amplified across various cancer types, as well as specific *BRAFV600E* mutations. Overall, CNVs, including both gains (amplifications) and losses (deletions), were detected in 97% (32/33) of patients in our cohort (Figure 1A). Among the total CNVs identified, 84% (169/201) were amplifications, and 16% (32/201) were deletions. The median number of CNVs per patient was 5 alterations, ranging from 0 to 18 CNVs. The most frequently amplified genes in ALM patients were *CCND1* (57.6%), *MYC* (48.5%), *CDK4* (39.4%), *FGFR1* (33.3%), *CCND2* (33.3%), *EGFR* (30.3%), *ERBB2* (27.3%), and *MDM2* (24.2%) (Figure 1A). The most frequently deleted genes were *RET* (12.1%) and *SMO* (9.1%). We found that 32.4% of the patients had one copy of the *AR* gene. This is because the *AR* gene is located on the X chromosome, and under normal conditions, males have only one copy of this gene. In our cohort, 80% (12/15) of the male patients have one copy of the gene, indicating no deletions in the *AR* gene. The *BRAFV600E* mutation was detected in 12.1% of patients (Figure 1A). Furthermore, the gene dosage of the total amplifications detected was mostly three copies (73.4%), while a lower proportion had four or more copies (26.6%). The genes with the highest gene dosage in the patient cohort were *CDK4* (30.3%), *CCND1* (24.2%), and *MDM2* (18.1%) (Figure 1B).

Next, we focused on the specific genes with the highest percentage of amplification in our cohort, as an increased copy number in these genes could have a greater impact on tumor initiation and progression. Specifically, we compared the presence (AMP) or absence (WT) of amplifications in *CCND1*, *MYC*, *CDK4*, *FGFR1*, *CCND2*, *EGFR*, *ERBB2*, and *MDM2* with the clinicopathological characteristics of our patient cohort (Supplementary Table S1). Additionally, we performed an MSS and DFS analysis according to these gene amplifications (Supplementary Tables S2 and S3, respectively).

Our results revealed that *EGFR^AMP^* was associated with higher-risk melanomas and reduced DFS (*p* = 0.049), while *MDM2^AMP^* correlated with worse MSS (*p* = 0.028) and DFS (*p* = 0.016). Interestingly, *CDK4^AMP^* patients significantly exhibited higher Breslow thickness (*p* = 0.010), presence of ulceration (*p* = 0.0092), greater mitotic index (*p* = 0.019), and were more likely to be diagnosed with high-risk melanomas (*p* = 0.037). In addition, patients with *CDK4^AMP^* showed worse MSS (*p* = 0.0208) and DFS (*p* = 0.0126) compared with *CDK4^WT^* patients (Supplementary Tables S1–S3), indicating a worse overall prognosis for these patients.

### 2.3. High CNVs in ALM Biopsies Are Associated with Worse Clinicopathological Characteristics and Prognosis

**Figure 2:**
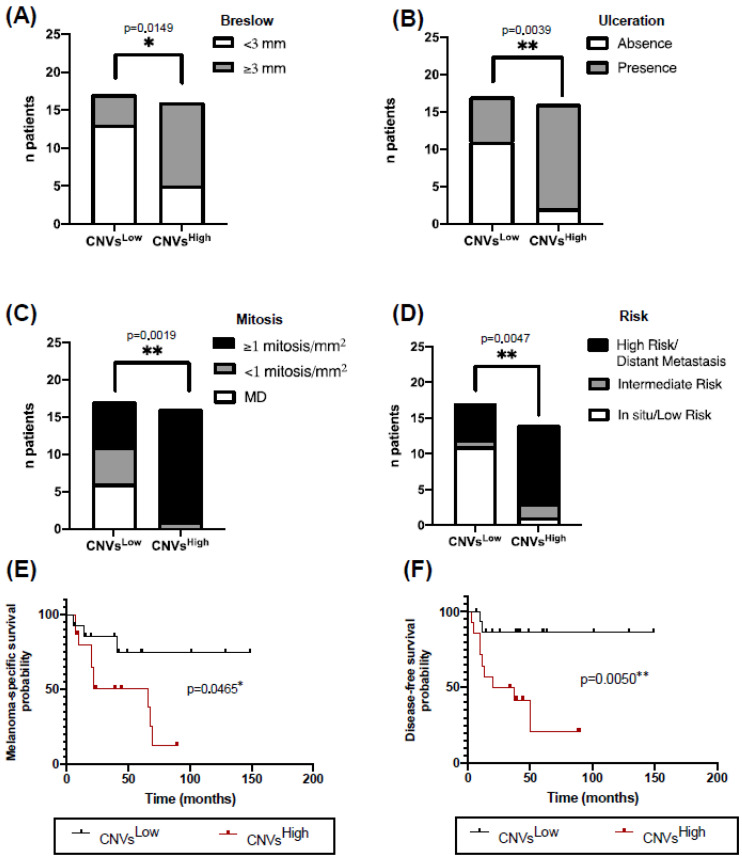
High copy number variation (CNV^High^) in ALM biopsies correlates with worse clinicopathological characteristics and poorer prognosis. Contingency analysis of (**A**) Breslow thickness, (**B**) presence or absence of ulceration, (**C**) mitotic index, and (**D**) risk associated with AJCC stage according to the total CNVs, classified as CNVs^Low^ (<5) or CNVs^High^ (≥5). Kaplan–Meier of (**E**) Melanoma-Specific Survival (MSS) and (**F**) disease-free survival (DFS) of ALM patients with CNVs^Low^ vs. CNVs^High^. Statistical analysis was performed using Fisher’s exact test for 2 × 2 contingency tables in (**A**–**C**), Pearson’s Chi-square test in (**D**) and Log-Rank test in (**E**,**F**) (* *p*-value < 0.05; ** *p*-value < 0.01).

Firstly, we grouped the patients into two groups based on the median of the total number of CNVs per patient (amplifications and deletions) as the cutoff point: low copy number variations (CNVs^Low^; CNVs ≤ 5) and high copy number variations (CNVs^High^; CNVs > 5). We then compared the clinicopathological characteristics between these groups of patients. CNVs^High^ group of patients predominantly presented thicker Breslow indices (≥3 mm; 11/16; *p* = 0.0149), higher presence of ulceration (14/16; *p* = 0.039), and higher mitotic index (15/16; *p* = 0.0019) compared with those in the CNVs^Low^ patients (Figure 2A–C). Moreover, the majority of patients with in situ/low-risk ALM were significantly distributed in the CNVs^Low^ group (11/12; *p* = 0.0047), whereas those with high-risk/distant metastasis were primarily found in the CNVs^High^ group (11/16) (Figure 2D). Interestingly, MSS and DFS analysis revealed that CNVs^High^ had a significantly worse outcome (MSS, *p* = 0.0465; DFS, *p* = 0.0050). Specifically, CNVs^High^ patients exhibited a mean MSS of 44.6 months (95% CI, 28.8–60.4 months) compared with 116.7 months (95% CI, 84.9–148.7 months) in the CNVs^Low^ group (Figure 2E). Similarly, the mean DFS was 37.2 months (95% CI, 18.5–56.0 months) for CNVs^High^ group vs. 130.6 months (95% CI, 106.9–154.3 months) for CNVs^Low^ group (Figure 2F).

### 2.4. Characterization of Copy-Number Variations in ALM Cell Lines

**Figure 3:**
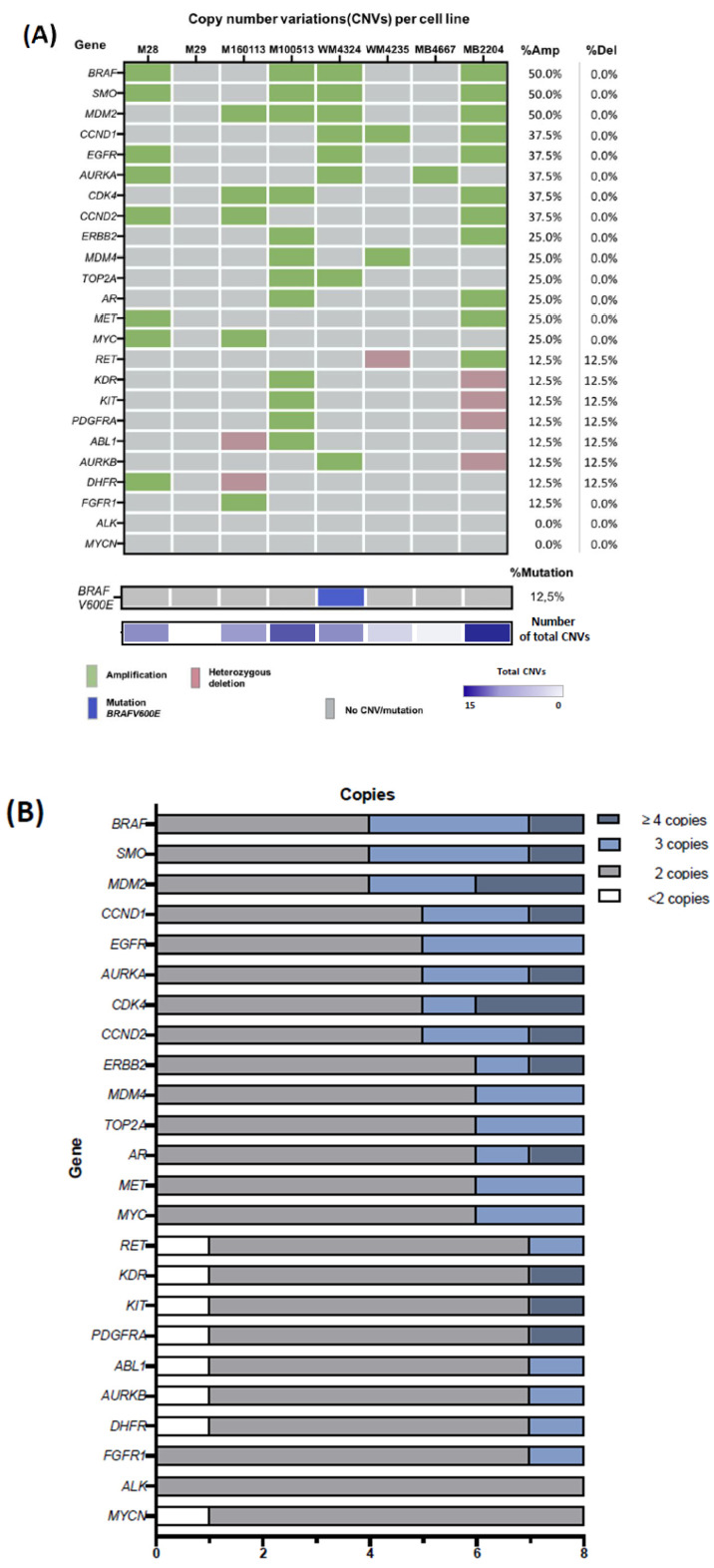
Copy number variations (CNVs) and *BRAFV600E* mutation analysis using Multiplex Ligation-dependent Probe Amplification (MLPA) in ALM cell lines. (**A**) Heatmap of CNVs identified in 24 oncogenes (up) and *BRAFV600E* mutation (bottom), along with the amplification and deletion frequency per gene (right). Genes are arranged in descending order according to the percentage of amplification. (**B**) Copy number quantification of each oncogene analyzed using MLPA. ALM cell lines are grouped according to the copy number in the following categories: <2 copies (deletion), 2 copies (diploid), 3 copies (gain of 1 copy), and ≥4 copies.

To develop an in vitro model in accordance with the results obtained from our patient cohort, we performed a pre-designed MLPA to identify CNVs in 24 oncogenes and the presence of the *BRAFV600E* mutation in eight human ALM cell lines, using the same approach as for patient biopsies. CNVs were detected in 87.5% (7/8) of ALM cell lines (Figure 3A). Out of all the CNVs identified, 87% (47/54) were amplifications, and the remaining 13% (7/54) were deletions. The median number of CNVs per cell line was 7 alterations, ranging from 0 to 15. The most frequently amplified genes were *BRAF* (50.0%), *SMO* (50.0%), *MDM2* (50%), *CCND1* (37.5%), *EGFR* (37.5%), *AURKA* (37.5%), *CDK4* (37.5%), and *CCND2* (37.5%). Genes with heterozygous deletions were *RET*, *KDR*, *KIT*, *PDGFRA*, *ABL1*, *AURKB,* and *DHFR*, all of them at a rate of 12.5%. When comparing CNV amplification between patient biopsies and the cell lines, several genes showed similar amplification percentages: *CCND1* (57.6% in biopsies vs. 37.5% in the cell line), *CDK4* (39.4% vs. 37.5%), *CCND2* (33.3% vs. 37.5%), *EGFR* (30.3% vs. 37.5%), and *ERBB2* (27.3% vs. 25%). However, *BRAF* (18.2% in biopsies vs. 50% in the cell line) and *MDM2* (24.2% vs. 50%) showed higher amplification rates in cell lines compared with patient ALM biopsies. Regarding gene dosage, most amplifications in cell lines involved three copies (70.2%), while a smaller proportion had four or more copies (29.8%). Genes with the highest gene dosage in overall cell lines were *CDK4*, *MDM2*, and CCND1 (each at 25%), close to the trend observed in the patient cohort (Figure 3B).

### 2.5. CNVs^High^ Cells Secretome Exhibits an Increased Pro-Tumor Profile

Given our observation that high CNVs are associated with poorer prognosis in ALM, we aimed to investigate whether this adverse prognosis could be linked to a more immunosuppressive TME. While many studies have explored TME-related signatures through multi-omic approaches, particularly using RNA-seq data to investigate immunosuppressive mechanisms, our study focused on analyzing the secretome of acral melanoma cells with immunosuppressive potential. Firstly, to assess whether CNVs could influence the secretome profile of ALM, cell lines were classified into two groups based on CNVs count, following the same criteria used for our ALM biopsies cohort (CNVs^Low^: CNVs < 5; CNVs^High^: CNVs ≥ 5). CNVs^Low^ cell lines were M29, WM4235, and MB4667 and CNVs^High^ cells included M28, M160113, M100513, WM4324, and MB2204 (Figure 3A).

**Figure 4:**
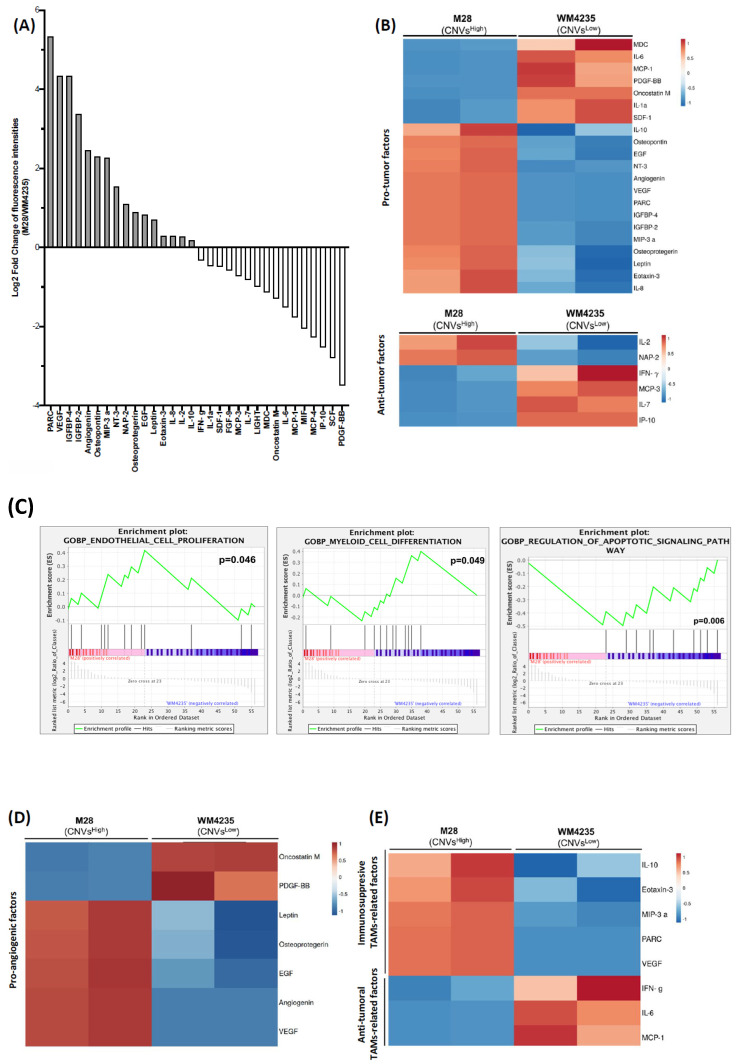
CNVs^High^ cells secretome exhibits a more pro-tumor, pro-angiogenic, and myeloid differentiation profile. (**A**) Log2 fold-change of fluorescence intensities obtained from the analysis of a cytokine detection array in the conditioned media (CM) from M28-CNVs^High^ vs. WM4235-CNVs^Low^ ALM cell lines. (**B**) Heatmaps of secreted factors in conditioned media (CM) from M28-CNVs^High^ vs. WM4235-CNVs^Low^ ALM cell lines, classified into pro-tumor factors (up) and anti-tumor secreted factors (down). (**C**) Gene set enrichment plots of endothelial proliferation, myeloid cell differentiation, and regulation of apoptotic signaling pathway obtained from a Gene Set Enrichment Analysis (GSEA) using the secretion data from ALM cell lines. (**D**) Heatmap of secreted factors in CM from M28-CNVs^High^ vs. WM4235-CNVs^Low^ ALM cell lines, classified into pro-angiogenic factors, and (**E**) anti-tumor or immunosuppressive TAMs-related factors.

We performed the secretome study from conditioned media (CM) of a representative CNVs^High^ ALM cell line (M28-CNVs^High^) and CNVs^Low^ cell line (WM4235-CNVs^Low^), using a human cytokine array consisting of 80 secreted factors (including cytokines, chemokines and growth factors). Out of the 58 factors detected, our results revealed significant differences in the secretion levels of 32 factors between ALM cell lines (Figure 4A and Supplementary Table S4). These factors were classified based on their pro-tumor or anti-tumor function within the tumor microenvironment (TME) [31,32,33,34,35,36,37,38,39,40,41,42,43]. M28-CNVs^High^ cells secreted increased levels of pro-tumor factors, such as PARC, VEGF, IL-10, and IL-8 and less secretion of anti-tumor factors (Figure 4B). To further explore our observations, we performed Gene Ontology (GO) enrichment analysis using Gene Set Enrichment Analysis (GSEA) based on the secretion profile of ALM cell lines. In CNVs^High^ cells secretome, we identified enrichment in biological processes related to endothelial cell proliferation and myeloid cell differentiation processes (Figure 4C). In contrast, CNVs^Low^ cells secretome was enriched for processes related to the regulation of the apoptotic signaling pathway (Figure 4C; Supplementary Table S5). According to these findings, we classified the cytokines into pro-angiogenic factors and those factors that polarize macrophages toward anti-tumoral or immunosuppressive phenotype. The cytokine array analysis showed increased secretion of pro-angiogenic and immunosuppressive TAMs-related factors in the CM of M28-CNVs^High^ cells compared with WM4235-CNVs^Low^ cells (Figure 4D,E). Additionally, PARC and VEGF exhibited the greatest fold-change (FC) increase in the CM of M28-CNVs^High^ cells. These secreted factors are known to play a pivotal role in pro-tumor progression, specifically contributing to immunosuppressive TAM polarization [44] and angiogenesis [32,45] (Figure 4A). Overall, despite the limitation resulting from the small number of cell lines analyzed, our data suggest that CNVs^High^ genetic profile is associated with a secretome enriched in pro-tumor factors, particularly those involved in promoting angiogenesis and the differentiation of immunosuppressive macrophages.

### 2.6. CNVs^High^ Secretome Induce Tumor-Promoting Macrophages Polarization and Enhanced Immunosuppressive TAMs at the Invasive Front of CNVs^High^ ALM Biopsies

**Figure 5:**
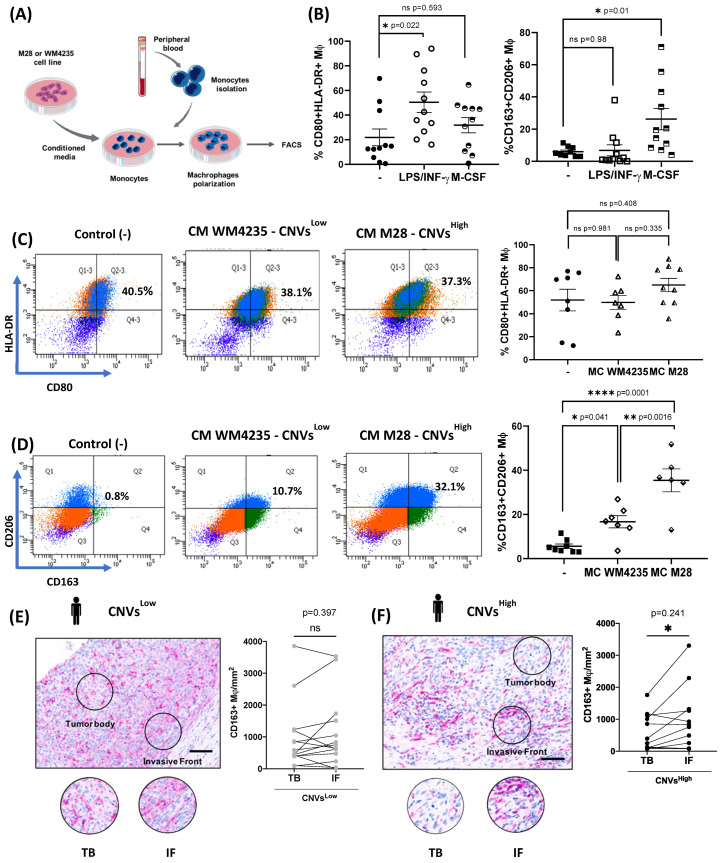
Secreted factors from CNVs^High^ cells promote immunosuppressive macrophage polarization. (**A**) Schematic workflow of the in vitro polarization procedure of macrophage polarization using CM from ALM cell lines. (**B**) Quantification (%) of CD80+HLA-DR+ (left) and CD163+CD206+ (right) macrophages after stimulation with LPS/IFN-g, M-CSF, or media only (-). Representative flow cytometry dot plots from one donor (left) and total quantification (right) of (**C**) %CD80+HLA-DR+ macrophages or (**D**) %CD163+CD206+ macrophages after treatment with CM from WM4235-CNVs^Low^, M28-CNVs^High^, or culture media only. To perform these experiments, we used n = 6 healthy donors. Invasive front of CNVs^High^ ALM biopsies are enriched in immunosuppressive TAM infiltration. (**E**) Representative images (left) and quantification (right) of CD163+ cells/mm^2^ in tumor body (TB) and invasive front (IF) within the same sample in CNVs^Low^ ALM biopsies (n = 14) and (**F**) CNVs^High^ ALM biopsies (n = 12). Scale bar: 100 μm. Statistical analysis was performed using One-Way ANOVA followed by Tukey’s post hoc test in (**A**–**D**) and paired t-test in (**E**,**F**) (ns, ns = not significant, * *p*-value < 0.05; ** *p*-value < 0.01; **** *p*-value < 0.0001).

Finally, we investigated whether there was a correlation between CNVs and macrophage polarization in ALM cells. To this end, we explored whether monocytes in vitro could differentiate into macrophages when exposed to CM from CNVs^Low^ and CNVs^High^ cells. We performed in vitro macrophage polarization through the treatment of monocytes with control cytokines (LPS/IFN-γ and M-CSF) and CM from ALM cells (Figure 5A). As expected, LPS/IFN-γ treatment increased HLA-DR^+^CD80^+^ (*p* = 0.040) expression in macrophages (anti-tumor macrophages) and M-CSF induced CD206^+^CD163^+^ (*p* = 0.0082) macrophages (immunosuppressive macrophages) (Figure 5B). CM from neither M28-CNVs^High^ cells nor WM4235-CNVs^Low^ cells promoted the expression of HLA-DR^+^CD80^+^ macrophages (*p* = 0.0833) compared with culture media alone (Figure 5C). However, both ALM cells CM induced CD206+CD163+ macrophage expression with a significantly higher induction in CM from M28-CNVs^High^ cells compared with CM from WM4235-CNVs^Low^ (*p* = 0.0016) (Figure 5D).

To extend these findings to our ALM patient cohort, we decided to explore the infiltration of immunosuppressive TAMs in ALM biopsies using IHC against CD163, a well-established marker of immunosuppressive macrophages. CD163+ cells were quantified in both the tumor body (TB) and the invasive front (IF) of each ALM biopsy. No regional differences in CD163+ expression were found in CNVs^Low^ ALM biopsies (n = 14; *p* = 0.2984) (Figure 5E). In contrast, CNVs^High^ ALM biopsies (n = 12) showed a significant increase in CD163+ TAMs at the IFs (*p* = 0.0397) (Figure 5F). Altogether, these data suggest that CNVs^High^ ALM cells exhibit an immunosuppressive secretome, contributing to a tumor-promoting microenvironment and potentially driving the polarization of immunosuppressive macrophages at the invasive front of ALM biopsies.

## 3. Discussion

ALM is a rare melanoma subtype associated with poor prognosis [2,46,47]. Due to its limited prevalence, few research analyses are available to elucidate the pathogenesis of this disease [12]. Therefore, multidimensional studies are essential to gain a profound understanding of ALM disease. In this study, we show a detailed characterization of the genetic signature of ALM patients, focusing on CNVs in relevant oncogenes that are commonly amplified across various cancers and their association with the clinicopathological characteristics of our patient cohort.

In our analysis focused on genetic profile, *CCND1* was the most amplified gene, followed by *MYC* and *CDK4*. Both *CCND1* and *CDK4* are well-established oncogenes in ALM, and both are involved in the G1 to S cell cycle transition [14]. In addition, *CCND2* and *MDM2* were among the most amplified genes in the cohort, both involved in the transition to the S phase of the cell cycle, suggesting the importance of this pathway in ALM pathogenesis and tumor evolution [48,49,50]. The *BRAFV600E* mutation was rare in our cohort, consistent with previous studies that reported a lower frequency of this mutation in ALM compared with other melanoma subtypes [3,5,48].

In this work, the association between genetics and clinical characteristics revealed that patients with CNVs^High^ exhibited more aggressive clinicopathological features and worse prognoses. While the relationship between total CNVs and poorer prognosis had not been previously established in ALM, similar associations have been described in other cancer types, where high CNVs correlate with metastasis and disease progression [51,52]. Additionally, it is noteworthy that none of the patients in our cohort diagnosed with in situ ALM presented CNVs, suggesting a possible association between CNVs^High^, tumor thickness, and disease progression. Amon all the oncogenes analyzed, *CDK4* amplification showed the most significant associations with clinicopathological characteristics and patient prognosis. Specifically, CDK4 amplification was linked to more aggressive tumor features—higher Breslow thickness, ulceration, mitotic rate, and clinical high-risk classification—as well as poorer MSS and DFS, underscoring its potential as a prognostic biomarker in ALM patients. According to our findings, recent studies have also identified *CDK4* amplification as an independent factor associated with reduced overall survival in primary acral melanoma patients [13].

Evaluating the genetic profile of ALM cell lines, we identified recurrent amplifications in several oncogenes, most notably *BRAF*, *MDM2*, *CCND1*, *CDK4*, and *CCND2*. Interestingly, the amplification of genes within the CDK4-CCND1/2 pathway, key regulators of the cell cycle, reflected the patterns observed in the patient cohort. Amplification frequencies of genes like *CCND1*, *CDK4*, *CCND2*, *EGFR*, and *ERBB2* were comparable between biopsies and cell lines; however, *BRAF* and *MDM2* amplifications were notably higher in cell lines. In addition, gene dosage amplification of three copies in ALM cell lines and the highest gene dosage, seen in *CDK4*, *MDM2*, and *CCND1*, closely matched the amplification trends observed in the patient cohort. The ALM cell lines used in this study derived from primary tumors or patient-derived xenografts (PDXs) and occasionally from metastatic lesions [53,54,55], supporting their relevance—despite some limitations—as models of the CNV landscape observed in clinical ALM samples.

To the best of our knowledge, the secretome of ALM cells in relation to the genetic profile had not been previously investigated as a relevant role in the immunomodulation of the TME. In this study, we investigated the ALM secretome within the context of ALM’s genetic profile, delving into its functional impact within the TME. CNVs^High^ cells exhibited a pro-tumoral and immunosuppressive secretome profile associated with gene subsets involved in angiogenesis and myeloid differentiation. Additionally, the secretome of the M28-CNVs^High^ cell line promoted in vitro polarization of macrophages toward an immunosuppressive phenotype. Previous studies reported that ALM patients presented a higher number of immunosuppressive macrophages compared with patients diagnosed with other cutaneous melanomas [56]. However, to our current knowledge, there are no previous studies that linked ALM secretome and tumor macrophage polarization to specific genetic profiles.

A recent study reported a decrease in anti-tumor macrophages at the IF compared with the TB of primary acral melanomas with *CDK4^AMP^* genetic profile [13]. In the present study, we found that patients with CNVs^High^ exhibited an increased number of CD163+ cells at the IF compared with the TB of ALM biopsies. In contrast, although CNVs^Low^ cells were still able to recruit and polarize TAMs to immunosuppressive phenotype, no significant regional differences in TAM infiltration were observed between TB and IF in these biopsies. This observation suggests that the increased presence of tumor-permissive macrophages in the IF may endorse an immunosuppressive microenvironment and could facilitate invasion and metastasis in the CNVs^High^ group of patients [57]. Additionally, a recent study has identified a specific molecular subtype of invasive acral melanoma (the “proliferation” phenotype, C3) characterized by high immune cell infiltration, including immunosuppressive APOE+/CD163+ macrophages. These macrophages are thought to play a pro-tumoral role, potentially leading to a worse prognosis [58].

In conclusion, this study explores the prognostic impact of ALM genetics and its relationship with the tumor secretome. Our findings demonstrate that a high number of CNVs correlates with poor prognosis in ALM patients. Our work also provides nice evidence for the positive association of CNVs^High^ cells with a pro-tumor secretome, promoting the polarization of immunosuppressive macrophages. Furthermore, we observed an increased percentage of immunosuppressive (CD163+) macrophages at the IF of CNVs^High^ ALM biopsies. While these results provide valuable prognostic insights, it is essential to consider the limitations of our study, notably the modest number of samples and cell lines analyzed from the precious cohort. Despite these constraints, the secretome emerges as a valuable tool for future diagnostic and monitoring applications in ALM patients. In particular, circulating cytokines detected in the plasma of patients may serve as prognostic biomarkers in CNVs^High^ patients, who may benefit from intensified clinical surveillance for early detection of metastatic dissemination. Furthermore, we would like to suggest that CNVs^High^ patients associated with worse prognosis and immunosuppressive TME have the potential benefit from adjuvant immunotherapy. Overall, our findings suggest new patients’ stratification based on genetic and TME secretome profiles to improve personalized treatment strategies and prognosis in ALM patients.

## 4. Materials and Methods

### 4.1. Cohort Construction and Patient Information

We conducted a retrospective review of patients diagnosed with ALM in the Dermatology Department of the Hospital Universitari Arnau de Vilanova (HUAV, Lleida, Spain) from 2008 to 2021. Patients were staged according to the 7th edition of the American Joint Committee on Cancer (AJCC) staging system. The clinicopathological information was registered, including age, sex, Breslow thickness, ulceration, mitotic index, AJCC stage and risk associated with the AJCC stage. Melanoma-Specific Survival (MSS) and Disease-Free Survival (DFS) were calculated until the last follow-up or death and until relapse (appearance of metastasis), respectively.

### 4.2. Patient Biopsies Collection

Thirty-three formalin-fixed paraffin-embedded (FFPE) biopsies were diagnosed as primary ALM, characterized by lentiginous proliferation of radial growth phase, by two pathologists from the Pathology Department of HUAV of Lleida. Samples were obtained with the support of the Xarxa de Bancs de Tumors de Catalunya, sponsored by Pla Director d’Oncología de Catalunya (XBTC), IRBLleida Biobank (B.0000682), and PLATAFORMA BIOBANCOS PT20/00021. Ethical approval was obtained from the Research Ethics Committee of HUAV (ref: CEIC-2230) with specific, informed consent, along with the Scientific Committee of the Biobank of IRBLleida.

### 4.3. ALM Cell Lines

Eight human ALM cell lines were used in this study. M28 and M29 cell lines were obtained from Dr. R. Vilella and Dr. S. Puig from Hospital Clinic-IDIBAPS (Barcelona, Spain); WM4235 and WM4324 cell lines were from Dr. M. Herlyn at The Wistar Institute (Philadelphia, PA, USA); MB4667 and MB2204 cell lines were from Dr. K. Couts at University of Colorado (Boulder, CO, USA). M160113 and M100513 cells from primary culture were obtained from Dr. M. Levesque at the Department of Dermatology, University Hospital Zürich (Zürich, Switzerland). Cell lines were grown in DMEM medium (Gibco, Waltham, MA, USA), and primary cell cultures were grown in RPMI 1640 medium (Gibco). Both media were supplemented with 10% heat-inactivated fetal bovine serum (FBS) (Gibco), 1% penicillin/streptomycin (p/s) (Gibco), and 0.1% Amphotericin B (Gibco). All cells were maintained at 37 °C with saturating humidity and 5% CO_2_.

### 4.4. Conditioned Media (CM) Collection

For the macrophage polarization procedure, ALM cells were seeded at 70% confluence and were grown in DMEM medium with 10% FBS and 1% p/s (10.000 U/mL penicillin and 10 mg/mL streptomycin (P/S; P0781, Sigma–Aldrich, St. Louis, MO, USA)). When cells reached 90% confluence, the medium was changed to RPMI with 2% FBS and 1% p/s and incubated for 24 h. Then, CM was collected, spun down to remove debris (14,000 rpm, 5 min, 4 °C), and stored at −80 °C.

### 4.5. DNA Extraction

Tissue was obtained from tumor regions of paraffin-embedded biopsies using microdissection techniques. DNA from FFPE biopsies was extracted by a Maxwell^®^ FFPE Plus DNA Kit (Promega, Madison, WI, USA) and was quantified by a NanoDrop ND 2000 (Thermo Fisher Scientific^®^, Waltham, MA, USA).

### 4.6. Multiplex Ligation-Dependent Probe Amplification (MLPA) Procedure

MLPA procedure was performed according to the manufacturer’s instructions using a pre-designed SALSA MLPA Probemix P175 Tumor Gain (MRC-Holland, Amsterdam, The Netherlands). The MLPA was designed to detect CNVs from 24 chromosomal loci of oncogenes, frequently amplified in various types of cancer, and *BRAFV600E* mutation. Amplified samples were analyzed by capillary electrophoresis on SeqStudio Genetic Analyzer (Thermo Fisher Scientific^®^). MLPA results were evaluated using Coffalyser.Net^TM^ software, https://www.mrcholland.com/technology/software, accessed on 20 September 2024 (MRC-Holland, Amsterdam, The Netherlands) with default settings. The relative CNV ratio of each ALM sample was calculated compared to non-tumor DNA as reference samples. These reference samples included control DNA provided by the MLPA kit, DNA extracted from normal skin biopsies of a patient and DNA isolated from the peripheral blood of a healthy donor. Significant differences were considered when the ratio was 0 (homozygous deletion), less than 0.7 (heterozygous deletion), or higher than 1.3 (amplification).

### 4.7. Immunohistochemistry (IHC)

IHC analyses were performed using CD163 antibody (1:100, Bio-Rad, Hercules, CA, USA, #MCA1853) according to the IHC Dako system (Glostrup, Denmark) [59]. CD163 staining was analyzed by quantifying the positive cell number per mm^2^ in the tumor body (TB) and the invasive front (IF) from whole biopsies. All analyses were performed by QuPath software version 0.5.1 [60].

### 4.8. Human Cytokine Array

Secreted media from M28 and WM4235 cell lines were collected and incubated in a Human Cytokine Antibody Array G5-8 (RayBiotech Life Inc., Peachtree Corners, GA, USA; AAH-CYT-G5-8) following the manufacturer’s instructions as described previously [61]. Glass chip was scanned with GenePix^®^ Microarray Scanner (Molecular Devices, LLC, San Jose, CA, USA) using Cy3 (excitation frequency = 532 nm). Results were analyzed with RayBio^®^ Analysis Tool, https://www.raybiotech.com/tools/array-analysis-tool, accessed on 20 September 2024. Log_2_Fold-Change and Z-score for each cytokine intensity value were calculated from both cell lines. Z-score was represented in heatmaps created by the web ClustVis tool, http://biit.cs.ut.ee/clustvis, accessed on 20 September 2024 [62]. The experiment was conducted using two independently collected conditioned media samples from each cell line.

### 4.9. Gene Set Enrichment Analysis

We performed a Gene Ontology (GO) enrichment of biological processes by Gene Set Enrichment Analysis (GSEA) [63] to identify the association between secreted factors detected by the Human Cytokine Array and gene signatures associated with biological processes. In GSEA analysis, we compared the data from M28 cells (high CNVs) vs. WM4235 cells (low CNVs) with adjusted *p* < 0.05.

### 4.10. Monocyte Isolation from Human Peripheral Blood

Peripheral blood was obtained from anonymized healthy volunteer donors with support from the IRBLleida Biobank (B.0000682) and PLATAFORMA BIOBANCOS PT20/00021. Peripheral blood mononuclear cells (PBMCs) were isolated by density gradient with Ficoll-Paque (Sigma-Aldrich^®^, St. Louis, MO, USA) after centrifugation at 400× *g* for 25 min. Afterward, we incubated PBMCs with RosseteSep Human CD3 depletion Cocktail (StemCell Technologies, Vancouver, BC, Canada; #15661) to deplete CD3+ cells. Recovered cells were washed twice in PBS 1X and we counted the total number of PBMCs and CD14+ positive cells using a mix of Perfect-count microspheres (Cytognos, Salamanca, Spain; #CY-PCM-50) with anti-CD14 antibody FITC (BD Biosciences, San Jose, CA, USA; #555397) by BD FACSCanto^TM^ II flow cytometer (BD Biosciences) [64]. Isolated human monocytes (5 × 10^6^ CD14+ cells each 24-well plate) were cultured with RPMI medium supplemented with 10% human AB serum (H4522, Sigma-Aldrich), 1% p/s (10.000 U/mL penicillin and 10 mg/mL streptomycin (P/S; P0781, Sigma-Aldrich)) at 37 °C and 5% CO_2_ incubator for 1 h approximately. Non-adherent cells were removed, and adherent cells were washed twice with PBS 1X and incubated with RPM1 1640 (Gibco, Waltham, MA, USA) supplemented with 10% heat-inactivated FBS and 1% p/s at 37 °C with saturating humidity and 5% CO_2_.

### 4.11. In Vitro Polarization of Macrophages

On day 1, monocytes were cultured with RPM1 1640 supplemented with 10% heat-inactivated FBS and 1% p/s at 37 °C and 5% CO_2_ incubator. On day 2, 50% well was replenished with the same volume of CM from ALM cell lines and cells were incubated for 72 h. LPS (200 ng/mL, Invivogen, San Diego, CA, USA) plus IFN-γ 100 ng/mL, Immunotools, Friesoythe, Germany) or M-CSF (400 ng/mL, Immunotools) were used as control stimuli of anti-tumor or immunosuppressive polarized macrophages, respectively.

On day 5, cells were washed with PBS and harvested with Accutase (Sigma-Aldrich^®^). Then, cells were incubated with blocking buffer (PBS 1X, 10% hAB serum, 2% FBS, 0.02% sodium azide) for 30 min at 4 °C. Cells were co-stained with CD80-APC (eBioscience^TM^, Thermo Fisher Scientific; #17-0809-42), HLA-DR-FITC (eBioscience^TM^, #11-9956-42), CD206-eFluor^TM^450 (eBioscience^TM^, #48-2069-42) and CD163-PE (eBioscience^TM^, #12-1639-42) for 20 min at 4 °C. Samples were washed with washing buffer (PBS 1X, 2% FBS, 0.02% sodium azide) twice and were fixed with 1% paraformaldehyde (PFA). Cells were resuspended in FACS Flow (BD Biosciences), acquired on a FACSCanto^TM^ II flow cytometer and analyzed using BD FACSDiva (BD Biosciences). The experiment was conducted with three independent replicates.

### 4.12. Statistical Analysis

Statistical analysis was performed using IBM SPSS Statistics^®^ 27.0 (IBM^®^, Armonk, NY, USA) and GraphPad Prism v9 (San Diego, CA, USA). Fisher’s exact test and Pearson’s Chi-square were used to evaluate categorical variables between groups. Melanoma-Specific Survival (MSS) and Disease-Free Survival (DFS) analyses were performed by the Log-Rank test. The means of two independent groups were analyzed by an unpaired t-test and the means of more than two independent groups were by a One-Way ANOVA followed by Tukey’s post hoc test. The means of the same patients were analyzed by a paired t-test. For column charts, error bars are the average ± SEM. *p*-values are indicated by asterisks * *p*-value < 0.05; ** *p*-value < 0.01; *** *p*-value < 0.001; **** *p*-value < 0.0001.

## Figures and Tables

**Table 1:** Clinicopathological characteristics of ALM patient cohort.

**Gender**	
Male	15 (45.5)
Female	18 (54.5)
**Age (years)**	
Mean (±SD)	70.2 (±15.9)
Median (IQR)	75 (54–83)
**Primary tumor location**	
**Hands n (%)**	**8 (24.2)**
Subungual	6 (18.2)
Finger	2 (6.1)
**Feet n (%)**	**25 (75.8)**
Subungual	8 (24.2)
Sole	11 (33.3)
Toe	3 (9.1)
Lateral	2 (6.1)
N/A	1 (3.0)
**Breslow thickness (mm)**	
Mean (±SD)	4.2 (±3.9)
Median (IQR)	2.7 (0.89–7.9)
**Ulceration n (%)**	
Yes	20 (60.6)
No	13 (39.4)
**Mitotic index n (%)**	
<1/mm^2^	6 (18.2)
≥1/mm^2^	21 (63.6)
N/A	6 (18.2)
**Live status n (%)**	
Alive	16 (48.4)
Melanoma death	13 (39.4)
Other causes	4 (12.1)
**Relapse (metastasis) n (%)**	
Yes	12 (36.3)
No	18 (54.5)
Distant metastasis at diagnosis	3 (9.1)

n = 33 patients. SD: Standard Deviation; IQR: Interquartile Range; N/A: Not Available.

## Data Availability

The original contributions presented in this study are included in the article and Supplementary Materials. Further inquiries can be directed to the corresponding authors.

## Supplementary Materials

The following supporting information can be downloaded at: https://www.mdpi.com/article/10.3390/ijms26094097/s1.
