# Supplementary material for: High Copy Number Variations Correlate with a Pro-Tumoral Microenvironment and Worse Prognosis in Acral Lentiginous Melanoma
Source: Int J Mol Sci. 2025 Apr 25;26(9):4097. doi: 10.3390/ijms26094097 (PMC12071846; doi:10.3390/ijms26094097)
Supplement: Supplementary file 1 [file ijms-26-04097-s001.zip › ijms-3566456-supplementary.pdf]

Supplementary Table S1. Contingency analysis of the genes with the highest number of amplifications detected by MLPA in relation to the clinicopathological characteristics of the ALM patient cohort.

		CCND1			*MYC*			*CDK4*			*FGFR1*			*CCND2*			*EGFR*			*ERBB2*			*MDM2*		
		WT	AMP	p-value	WT	AMP	p-value	WT	AMP	p-value	WT	AMP	p-value	WT	AMP	p-value	WT	AMP	p-value	WT	AMP	p-value	WT	AMP	p-value
Gender	Male	5	10	0,722	9	6	0,491	7	8	0,083	9	5	0,991	11	4	0,712	9	6	0,488	9	6	0,239	10	5	0,418
	Female	8	10		8	10		14	4		11	6		11	7		13	5		15	3		15	3	
Age(years)	≤75	8	9	0,481	12	5	0,038*	9	8	0,282	11	5	0,381	13	4	0,282	11	6	>0,99	12	5	>0,99	13	4	>0,99
	>75	5	11		5	11		12	4		9	6		9	7		11	5		12	4		12	4	
Breslow (mm)	<3	9	9	0,284	12	**6**	0,084	15	3	0,010*	12	5	0,732	14	4	0,138	15	3	0,061	15	3	0,239	15	3	0,418
	≥3	4	11		5	10		6	9		8	6		8	7		7	8		9	6		10	5	
Ulceration	Yes	7	6	0,171	8	12	0.157	9	11	0.009**	10	8	0,232	12	8	0,456	11	9	0,132	12	8	0,056	14	6	0,431
	No	6	14		9	4		12	1		10	3		10	3		11	2		12	1		11	2	
Mitotic index	<1 mitosis/mm^2	3	4	>0,99	3	4	>0,99	6	1	0,019*	5	2	0,419	4	3	>0,99	5	2	0,678	5	2	>0,99	6	1	0,633
	≥1 mitosis/mm^2	7	13		9	11		9	11		9	9		12	8		12	8		13	7		13	7	
Location	Subungual	10	5	0,003**	9	6	0,491	9	6	0,692	1	11	0,33	10	5	>0,99	12	3	0,266	13	2	0,134	14	4	>0,99
	Other	3	15		8	10		12	6		1	9		12	6		10	8		11	7		11	4	
Risk	In situ/Low Risk	7	5	0,239	9	3	0,07	10	2	0,037*	9	3	0,584	10	2	0,287	11	1	0,048*	11	1	0,178	11	1	0,273
	Intermediate Risk	1	2		2	1		3	0		2	1		2	1		2	1		2	1		2	1	
	High Risk/Distant Metastasis	5	13		6	12		8	10		9	7		10	8		9	9		11	7		12	6	

Supplementary Table S2. MLA-specific survival analysis according to the presence of amplification in genes with the highest number of amplifications detected by MLPA.

		Patients (n)	% Melanoma-specific survival 1 year (±SEP)	% Melanoma-specific survival 3 year (±SEP)	Chi-square (Log-Rank test)	p-value
CCND1	**WT**	13	92(±8)	63(±15)	0,351	0,533
	**AMP**	16	81(±10)	68(±12)		
MYC	**WT**	15	86(±9)	69(±13)	0,021	0,885
	**AMP**	14	86(±9)	63(±13)		
CDK4	**WT**	17	94(±6)	82(±10)	5,345	**0,021***
	**AMP**	12	73(±13)	39(±16)		
FGFR1	**WT**	17	82(±9)	60(±13)	0,951	0,621
	**AMP**	10	90(±9)	70(±14)		
CCND2	**WT**	19	83(±9)	70(±11)	0,895	0,344
	**AMP**	10	90(±9)	60(±15)		
EGFR	**WT**	19	83(±9)	77(±10)	2,181	0,14
	**AMP**	10	90(±9)	48(±16)		
ERBB2	**WT**	21	80(±9)	69(±11)	0,014	0,906
	**AMP**	8	100(±0)	60(±18)		
MDM2	**WT**	21	90(±7)	79(±9)	4,813	**0,028***
	**AMP**	8	73(±16)	29(±17)		

Supplementary Table S3. Disease-free survival analysis according to the presence of amplifications in genes with the highest number of amplifications detected by MLPA

		Patients (n)	% Disease-free survival 1 year (±SEP)	% Disease-free survival 3 year (±SEP)	Chi-square (Log-Rank test)	p-value
CCND1	**WT**	12	83(±11)	73(±13)		
	**AMP**	18	83(±13)	66(±11)	0,496	0,481
MYC	**WT**	16	87(±9)	66(±12)		
	**AMP**	14	79(±11)	71(±12)	0,141	0,708
CDK4	**WT**	20	95(±5)	79(±9)		
	**AMP**	10	58(±16)	46(±16)	6,226	**0,0126***
FGFR1	**WT**	19	78(±10)	60(±12)		
	**AMP**	9	89(±10)	78(±14)	1,346	0,51
CCND2	**WT**	21	85(±8)	69(±10)		
	**AMP**	9	78(±14)	67(±16)	1,439	0,23
EGFR	**WT**	21	85(±8)	80(±9)		
	**AMP**	9	78(±14)	44(±17)	3,884	**0,049***
ERBB2	**WT**	22	86(±7)	71(±10)		
	**AMP**	8	75(±15)	63(±17)	0,578	0,447
MDM2	**WT**	22	95(±5)	80(±9)		
	**AMP**	8	50(±18)	38(±17)	5,782	**0,016***

Supplementary Table S4. Analysis of soluble factors detected using the cytokine array according to the total CNVs (Copy Number Variations).

Cytokine	Z-score M28	Z-score WM4235	p-valor	Cytokine	Z-score M28	Z-score WM4235	p-valor
**Angiogenin**	0,87	-0,87	**0,0003*****	**IL-7**	-0,86	0,86	**0,0020****
**BDNF**	-0,54	0,54	0,3779	**IL-8**	0,85	-0,85	**0,0137***
**BLC**	-0,8	0,8	0,0763	**IP-10**	-0,87	0,87	**0,00002*****
**EGF**	0,86	-0,86	**0,0063****	**Leptin**	0,84	-0,84	**0,0263***
**Eotaxin-3**	0,85	-0,85	**0,0225***	**LIF**	-0,73	0,73	0,1547
**FGF-6**	-0,15	0,15	0,8218	**LIGHT**	-0,86	0,86	**0,0058****
**FGF-7**	0,6	-0,6	0,3047	**MCP-1**	-0,85	0,85	**0,0134***
**Flt-3 Ligand**	-0,54	0,54	0,3709	**MCP-3**	-0,86	0,86	**0,0027****
**GCP-2**	-0,81	0,81	0,0661	**MCSF**	0,35	-0,35	0,5945
**GCSF**	-0,73	0,73	0,1539	**MDC**	-0,83	0,83	**0,0404***
**GDNF**	-0,58	0,58	0,3338	**MIF**	-0,86	0,86	**0,0074****
**GM-CSF**	-0,54	0,54	0,3814	**MIG**	0,56	-0,56	0,3533
**GRO**	0,82	-0,82	0,0529	**MIP-3 a**	0,86	-0,86	**0,0014****
**GRO-a**	-0,13	0,13	0,8467	**NAP-2**	0,86	-0,86	**0,0031****
**IFN- g**	-0,83	0,83	**0,0376***	**NT-3**	0,87	-0,87	**0,0010****
**IGF-I**	-0,45	0,45	0,4792	**Oncostatin M**	-0,87	0,87	**0,0001*****
**IGFBP-2**	0,87	-0,87	**0,0010****	**Osteopontin**	0,86	-0,86	**0,0039****
**IGFBP-3**	-0,8	0,8	0,0786	**Osteoprotegerin**	0,85	-0,85	**0,0202***
**IGFBP-4**	0,87	-0,87	**0,0003*****	**PARC**	0,87	-0,87	**0,0002*****
**IL-1 b**	-0,8	0,8	0,0787	**PDGF-BB**	-0,86	0,86	**0,0110***
**IL-10**	0,83	-0,83	**0,0397***	**RANTES**	0,77	-0,77	0,1093
**IL-12 p70**	0,48	-0,48	0,447	**SDF-1**	-0,86	0,86	**0,0057****
**IL-13**	0,8	-0,8	0,0802	**TGF- b 2**	-0,81	0,81	0,0698
**IL-15**	-0,7	0,7	0,1914	**TGF-b1**	-0,44	0,44	0,4947
**IL-1a**	-0,86	0,86	**0,0058****	**Thrombopoietin**	-0,27	0,27	0,6909
**IL-2**	0,84	-0,84	**0,0331***	**TIMP-1**	-0,3	0,3	0,6494
**IL-3**	-0,68	0,68	0,2134	**TNF-a**	-0,18	0,18	0,7922
**IL-5**	-0,63	0,63	0,2728	**TNF-b**	0,33	-0,33	0,6163
**IL-6**	-0,86	0,86	**0,0031****	**VEGF**	0,87	-0,87	**0,0002*****

Supplementary Table S5. Top 20 Gene Set Enrichment Analysis (GSEA) results for Gene Ontology of Biological Process (GOBP) obtained from the analysis of a cytokine array with conditioned media from ALM cell lines.

Enriched in M28 conditioned medium			
NAME	SIZE	Enrichment Score (ES)	Nominal p-value (NOM p-val)
GOBP_ENDOTHELIAL_CELL_PROLIFERATION	12	0.4166667	0.045889102*
GOBP_MORPHOGENESIS_OF_A_BRANCHING_STRUCTURE	7	0.4971429	0.043392505*
GOBP_MYELOID_CELL_DIFFERENTIATION	12	0.4	0.048732944*
GOBP_POSITIVE_REGULATION_OF_ENDOTHELIAL_CELL_PROLIFERATION	8	0.46428573	0.056818184
GOBP_ACTIVATION_OF_PROTEIN_KINASE_ACTIVITY	7	0.49428576	0.05836576
GOBP_MYELOID_LEUKOCYTE_DIFFERENTIATION	11	0.39130437	0.085192695
GOBP_SKELETAL_SYSTEM_DEVELOPMENT	7	0.46000007	0.09496124
GOBP_B_CELL_MEDIATED_IMMUNITY	5	0.5	0.12256809
GOBP_ACTIN_FILAMENT_BASED_PROCESS	9	0.3958333	0.16122448
GOBP_REGULATION_OF_B_CELL_MEDIATED_IMMUNITY	5	0.5	0.1319149
GOBP_REGULATION_OF_INNATE_IMMUNE_RESPONSE	5	0.5	0.14845361
GOBP_TYPE_II_INTERFERON_PRODUCTION	6	0.46078432	0.14648438
GOBP_POSITIVE_REGULATION_OF_HYDROLASE_ACTIVITY	13	0.3339161	0.1570248
GOBP_RESPONSE_TO_XENOBIOTIC_STIMULUS	6	0.46078432	0.16155419
GOBP_REGULATION_OF_ANIMAL_ORGAN_MORPHOGENESIS	6	0.46078432	0.16237624
GOBP_REGULATION_OF_MYELOID_CELL_DIFFERENTIATION	9	0.375	0.15811089
GOBP_MULTI_MULTICELLULAR_ORGANISM_PROCESS	7	0.4114286	0.17669173
GOBP_GLAND_DEVELOPMENT	8	0.38520408	0.20464136
GOBP_G_PROTEIN_COUPLED_RECEPTOR_SIGNALING_PATHWAY	13	0.31118882	0.19845857
GOBP_NEGATIVE_REGULATION_OF_DEFENSE_RESPONSE	5	0.46153846	0.20436507

Enriched in WM4235 conditioned medium			
**Name**	**Size**	**Enrichment Score (ES)**	**Nominal p-value (NOM p-val)**
GOBP_REGULATION_OF_APOPTOTIC_SIGNALING_PATHWAY	10	-0.49574465	0.005859375**
GOBP_REGULATION_OF_LYMPHOCYTE_CHEMOTAXIS	6	-0.5980392	0.02268431*
GOBP_NEGATIVE_REGULATION_OF_APOPTOTIC_SIGNALING_PATHWAY	10	-0.49574465	0.020408163*
GOBP_POSITIVE_REGULATION_OF_CELL_DIFFERENTIATION	25	-0.36625	0.045454547*
GOBP_APOPTOTIC_SIGNALING_PATHWAY	14	-0.41694352	0.018442623*
GOBP_MESENCHYMAL_CELL_DIFFERENTIATION	5	-0.5769231	0.049212597*
GOBP_CELL_CELL_SIGNALING	35	-0.36103898	0.053149607
GOBP_MESENCHYME_DEVELOPMENT	5	-0.5769231	0.056
GOBP_NEGATIVE_REGULATION_OF_LOCOMOTION	8	-0.46938774	0.074
GOBP_CELLULAR_LIPID_METABOLIC_PROCESS	5	-0.54230773	0.0875
GOBP_REGULATION_OF_CELL_POPULATION_PROLIFERATION	44	-0.37062937	0.078470826
GOBP_REGULATION_OF_LYMPHOCYTE_MIGRATION	8	-0.4642857	0.07874016
GOBP_TAXIS	29	-0.31034487	0.068136275
GOBP_SIGNAL_RELEASE	13	-0.3741259	0.077220075
GOBP_DEFENSE_RESPONSE_TO_SYMBIONT	6	-0.4803922	0.08806262
GOBP_GLIOGENESIS	9	-0.4166667	0.07949791
GOBP_POSITIVE_REGULATION_OF_CHEMOKINE_PRODUCTION	6	-0.4803922	0.10139165
GOBP_REGULATION_OF_EXTRINSIC_APOPTOTIC_SIGNALING_PATHWAY	7	-0.45999998	0.09090909
GOBP_RESPONSE_TO_MOLECULE_OF_BACTERIAL_ORIGIN	23	-0.3094629	0.1090535
GOBP_POSITIVE_CHEMOTAXIS	10	-0.39999995	0.11016949
